# Notes and News

**Published:** 1891-03-28

**Authors:** 


### Notes and News.

COLLECTED AND COMMUNICATED.

With reference to the paper lately read by Mr. L. S. Bristowe, M.A. (of Lincoln's Inn), before the Hospitals' Association, on "The Legal Restrictions on Gifts to Charity," it has been resolved by the Council of the Hospitals' Association to draft a short Bill for presentation to Parliament, with a view to removing the disabilities at present existing.

The late Mr. Andrew Pickard bequeathed £1,000 each to the Leeds Infirmary, Wakefield Infirmary, and Dewsbury Infirmary, and £250 to the Leeds Hospital for Women and Children, besides large sums to other charities. The late Mrs. Jane Entwistle has left £200 each to the Tamworth Cottage Hospital, Surgical Aid Society, Birmingham Queen's, Women's, and Eye Hospitals, the National Hospital, and the Great Ormond Street Children's Hospital.

The report for the past year of the Durham County Hospital and Samaritan Society is both interesting and satisfactory. A third nurse has now been engaged, as the work had become too much for two, and a new nurses' home has been acquired. The report states that even small quantities of cod liver oil or malt extract, such as people frequently waste, would be considered a valuable gift by this charity. We hope in future these half bottles may find their way to Miss Haythorne, the Matron.

The subscribers to the Leicester Infirmary can have the satisfaction of knowing, down to the smallest detail, exactly how the funds have been administered. The annual report is one of the fullest and most complete which has come to hand. During the past year the Children's Hospital had no fewer than 477 little patients under treatment. This important and interesting addition to the work of the Infirmary entails a very serious increase in the annual expenditure; the Board, therefore, finds it necessary to make an urgent appeal for additional annual subscriptions.

The Prince of Wales presided at a meeting of the general committee of the Seventh International Congress of Hygiene and Demography, which had been convened to receive the reports of the various sub-committees, at the room of the Medical Society, Hanover Square. Sir Douglas Dalton, the Chairman of the organising committee, read a comprehensive report, giving the action which had been taken at home and abroad, in the Colonies and in India. The various reports having been read, their adoption was moved by Sir Henry Roscoe, M.P., who spoke of the success which was assured to the Congress, by the patronage of the Queen and the presidency of the Prince of Wales. In supporting the motion, which was seconded by Dr. George Buchanan, the Lord Provost of Edinburgh dwelt strongly on the efforts in the way of hygiene and sanitation, made by Edinburgh of late years. In replying to a vote of thanks, the Prince said he was sure that this Congress, the seventh of its series, will prove as successful as, if not more successful than any of its predecessors.

An institution which well deserves the attention of the charitable is the Glasgow Dental Hospital. It deals with and alleviates a very large amount of acute suffering among the poor of the city. It is satisfactory also to note that there is a growing proportion of preservative operations as compared with the cases of extraction, showing that the work of the hospital is devoted not only to the removal of pain, but to its prevention. The finances of the hospital have improved during 1890, owing chiefly to an increase in the sum derived as fees from students attending the hospital.

The report for 1890 of the York County Hospital, shows that the present financial position of the institution is most satisfactory. The sum of £2,792 has been invested during the year, and there still remained on December 31st, a balance in the hands of the treasurer of £80 14s. 10d. The in-patients treated during the year numbered 1,074, as against 1,374 in 1889, and the out-patients 8,198, as against 10,380, in 1889. With reference to this decrease in the number of patients, the Committee thank their subscribers for the increased discrimination used in distributing hospital notes, and their efforts in selecting only suitable cases.

The foundation-stone of the new infirmary of the West Derby Union was laid by Mr. John Ellison. The following is a description of the building: The main building on the north side of Mill Road contains the administrative block and five large blocks in addition to the lower hospital, which it is proposed to leave standing. The several floors of the blocks contain a large ward 108 feet by 24 feet, capable of accommodating 36 patients, duty room, and day room. A nurses' home is in a separate building. The building will be of grey brick with terra-cotta facings. The architect is Mr. C. H. Lancaster.

At St. Mary's Hospital, Paddington, the annual meeting of the Governors and subscribers was held on the 20th inst., Mr. J. Derby Allcroft presiding. From the annual report read by the active Secretary (Mr. Thomas Ryan), it appears that the hospital has performed an exceptionally heavy year's work, the number of patients relieved, both in the wards and in the out-patients' department, being higher than in any previous year. No less than 3,878 in-patients were treated. Considerable progress has been made during the year in buying up the various interests concerned in the property in Praed-street, the leasehold reversion of which was purchased from the Grand Junction Canal Company in 1888, to enable an increase to be made in the size of the hospital, and to give it a frontage on the main thoroughfare. The reason for the delay is to be found in the fact that exorbitant prices have been demanded in some quarters; but the Governors, while keenly alive to the necessity for carrying this most important work forward, do not think that time is of such vital consequence as to justify them in securing immediate possession at any price. In this we are sure all will agree. The meeting on Friday was more largely attended than has been the case for years. Special mention was made of the increase in the number of maternity out-patients, this department having doubled in extent within the last four years. In consequence of the exceptionally heavy year's work the total expenditure amounted to £23,608, and but for the receipt of upwards of £8,200 in legacies, the resources of the charity would have been unequal to the strain put upon them. The report was adopted on the motion of the Chairman, seconded by the Rev. Canon Duckworth. Among those present at the meeting were Sir Edward Sieveking, Admiral Sir E. Ommaney, and Dr. Farquharson, M.P.

FROM Chicago we learn that a man has been discovered who sheds his skin yearly; he peels it off his hands first as though he were taking off his gloves. Here is a development which the author of "Elsie Venner" missed, and we recommend it to our English writers of shilling shockers, who love to dwell on snaky subjects.

AT last there seems some prospect that the Acts protecting wild birds will receive their proper supplement in an Act for the protection of wild birds' eggs. The irrepressible boy (and who that is worth anything has not been an irrepressible boy?) is, as recent events have shown, not the only offender. But as for boys, Lord Lilford has wisely suggested that, if we were to teach them the rudiments of ornithology in such a manner as to make the study of natural history a pleasure, we should be in a better position to cure or, at least, alleviate the pernicious propensity which every boy has to throw a stone at every bird within his ken or to take all the eggs he may find in a nest.

THE authorities of the Royal Free Hospital have decided to have a festival dinner this year, in connection with the appeal for funds to rebuild the fourth and last side of the hospital. Lord Lathom will take the chair on this occasion, the date of which is May 2nd. It is some years since there has been a festival dinner in connection with this hospital, and as it has special claims on the public sympathy in that it was the first institution to establish the free principle, and also the first to admit women students to its wards, we hope that the appeal now in circulation may receive the prompt and liberal recognition which it merits.

THE Duke of Fife presided at the annual festival dinner of the Hospital for Sick Children, Great Ormond Street, held at the Hotel Metropole on the 18th inst. There was a large number of guests present, amongst whom were the Lord Mayor, Sir Charles Hall, Q.C., M.P., Sir Richard Quain, Viscount Gort, Mr. Justice Kekewich, the Hon. Evelyn Hubbard, Alderman Sir Polydore De Keyser, and Mr. Sheriff Augustus Harris. Though the hospital has only been established forty years, it was the first institution of its kind in Great Britain, and during its term of existence something like 520,000 patients have been treated. The average number of patients annually treated within its walls is about 60,000, and of these about 1,060 are in-patients; 6,371 new patients, on an average, are annually relieved. The annual expenditure amounts to about £11,000, to meet which the annual subscriptions only reach the small sum of £3,000. Funds are sadly needed to enable the hospital to complete the new wing, and so to enable them to carry out the good work on a still more extended scale. During the evening subscriptions in connection with the dinner, amounting to £4,300, were announced by the Secretary, Mr. Adrian Hope.

THE annual meeting of the Hampstead Home Hospital was held last Saturday, when Mr. Owthwaite, the secretary, presented a good report. There are twenty beds in this pretty little building on the northern heights of London, and private patients are taken in and carefully nursed and tended, getting all the advantages of a hospital without losing the feeling of home. Many of the patients have shown in a substantial manner their appreciation of the comfort and attention they experienced during their residence in the hospital; and one gentleman most kindly presented the hospital with a handsome chamber organ, and in addition recently handed the Sister Superintendent a cheque for £30, with the request that it be applied to the purchase of any useful article of furniture likely to add to the comfort of the nurses; he likewise subscribed five guineas to Mudie's Library for their benefit. The finances of the hospital are looking up, chiefly owing to the large subscriptions received at the festival dinner held a year ago. It is all very well for the "ower guid" to say that charities musn't beg or have bazaars or dinners, but are not these small sins better than the bigger one of getting into debt?

